# Surface Patterning of Gold Nanoparticles on PEG-Based Hydrogels to Control Cell Adhesion

**DOI:** 10.3390/polym9050154

**Published:** 2017-04-26

**Authors:** Fang Ren, Cigdem Yesildag, Zhenfang Zhang, Marga C. Lensen

**Affiliations:** 1 Nanopatterned Biomaterials, Technische Universität Berlin, Sekr. TC 1, Strasse des 17. Juni 124, Berlin 10623, Germany; renfang_ark@hotmail.com (F.R.); cigdem.yesildag@tu-berlin.de (C.Y.); 2 State Key Laboratory of Chemical Resource Engineering, Beijing University of Chemical Technology, Beijing 100029, China

**Keywords:** poly(ethylene glycol) hydrogels, gold nanoparticles, micro-contact deprinting, pattern transfer, cell adhesion

## Abstract

We report on a versatile and easy approach to micro-pattern gold nanoparticles (Au NPs) on 8-arm poly(ethylene glycol)-vinyl sulfone thiol (8PEG-VS-SH) hydrogels, and the application of these patterned Au NPs stripes in controlling cell adhesion. Firstly, the Au NPs were patterned on silicon wafers, and then they were transferred onto reactive, multifunctional 8PEG-VS-SH hydrogels. The patterned, micrometer-sized Au NPs stripes with variable spacings ranging from 20 μm to 50 μm were created by our recently developed micro-contact deprinting method. For this micro-contact deprinting approach, four different PEG-based stamp materials have been tested and it was found that the triblock copolymer PEG-PPG-PEG-(3BC) stamp established the best transfer efficiency and has been used in the ongoing work. After the successful creation of micro-patterns of Au NPs stripes on silicon, the patterns can be transferred conveniently and accurately to 8PEG-VS-SH hydrogel films. Subsequently these Au NPs patterns on 8PEG-VS-SH hydrogels have been investigated in cell culture with murine fibroblasts (L-929). The cells have been observed to adhere to and spread on those nano-patterned micro-lines in a remarkably selective and ordered manner.

## 1. Introduction

In recent decades, due to the unique electronic and photonic properties as well as easy functionalization, gold nanoparticles (Au NPs) have been utilized in physical [1,2] and biomedical fields [3,4,5]. With the increasing development of nanotechnology, the ability to generate patterns of Au NPs on substrate is important for biosensors [6], optics [7], catalysis [8] and biomaterial applications [9].

To control cell behavior by designing the appropriate environment is very important to understand biological systems [10]. Control of cell adhesion is crucial for tissue engineering and fundamental studies in cell biology like cell–cell, cell–substrate and cell–medium interactions [11]. Biomaterials modified by cell recognition motives (e.g., fibronectin protein) can be used to control the interaction between cells and synthetic substrates. Besides biochemical functionalization, cell behavior can be influenced by the stiffness, nanoscale topography and microscopic surface patterning of the substrate [12,13,14,15]. For example, Ding et al. showed that micro-patterns of peptide via micro-patterned Au NPs enable cell localization on the background of poly(ethylene glycol) (PEG) hydrogels [16]. It is highly interesting to assemble nanoparticles to create desired regions for cell adhesion [11,14,17]. 

As research continues, many methods to pattern nanoparticles have been successfully developed, by us and others [14,18]. All these methods can be generally categorized as “top-down” and “bottom-up” methods. However, some of them are time consuming and require expensive devices, and the obtained patterns consist of unordered, agglomerated, or densely compacted Au NPs.

Most efforts in cell micro-patterning have focused on micro-fabrication techniques that are based on silicon or glass substrates, which limit applications to tissue engineering. PEG hydrogels, which possess an inert and protein-repellent surface have demonstrated to be especially useful as a background platform for the in vitro investigation of cell behavior, when applied in biosensor systems and tissue engineering [19,20]. In this contribution, we report a novel technique to pattern regularly arranged Au NPs on silicon wafers, and the Au NPs patterns can be transferred to the 8PEG-VS-SH hydrogel by a “micro-contact deprinting method” [18]. These micro-patterned Au NPs composite hydrogels enable us to control cell adhesion of murine fibroblasts L-929 in an ordered way following the patterned Au NPs stripes.

Hydrogels are crosslinked hydrophilic polymer networks. The presence of water in gel matrix provide hydrogels with a high porosity, a large surface area, softness, flexibility and biocompatibility [19,21,22]. PEG hydrogels are among the most widely studied and extensively used polymers as matrix for controlling drug delivery, as well as cell delivery vehicles for promoting tissue regeneration. The network properties, swelling and the elasticity of the gels can be controlled by tuning the length of polymers and their functionalities. In addition, PEG hydrogels are optically transparent, allowing effective optical detection with minimal background signals [23,24]. Moreover, the properties of preventing non-specific protein adsorption and undesired cell attachment also make it a perfect cell-resistant substrate for biomaterial investigation [25]. Thus, in this work, PEG hydrogels have been chosen as the basic material to template the immobilization of Au NPs. Very recently, we discovered that the presence of non-functionalized Au NPs on cell-anti-adhesive PEG hydrogels enabled cell adhesion [14]. In recent literature, it was shown that also carbon nanotubes or nano-whiskers can improve cell adhesion and control orientation and even differentiation [26,27].

Au NPs can be immobilized onto hydrogels by entrapment, chemical adsorption or physical adsorption [28]. Physical adsorption method is one of the most common methods for synthesis of nanocomposite hydrogels by mixing nanoparticles with precursors of hydrogels or modifying Au NPs on the surface of hydrogel surface [29,30]. As the development of bio-conjugate chemistry and nanotechnology progresses, immobilization of Au NPs can be achieved via highly specific biomolecular interactions. In this study, both physical adsorption and covalent linkages have been adopted to immobilize Au NPs onto hydrogel surfaces. Au NPs were firstly deposited on (3-Aminopropyl) triethoxysilane (APTES)-modified silicon wafers through the electrostatic interaction between the positively charged amino groups of APTES and negative charges on the citrate-stabilized Au NPs. Secondly, the hydrogels were brought into conformal contact with the surface of the Au NPs-decorated silicon wafers. Lastly, the immobilization of Au NPs on the hydrogels was achieved by peeling off the hydrogels from silicon wafers. By this procedure, Au NPs are effectively and quantitatively transferred from the silicon wafers to the PEG-hydrogel surface. 

## 2. Materials and Methods

### 2.1. Materials

Isopropanol, acetone, ammonia (25%), hydrogen peroxide (H_2_O_2_ 30%), concentrated sulfuric acid (H_2_SO_4_ 98%) and toluene were purchased from Carl Roth (Karlsruhe, Germany). (3-aminopropyl) triethoxysilane (APTES) was from ABCR GmbH (Karlsruhe, Germany), Acryloyl chloride, 2-iminothiolane hydrochloride, vinyl sulfone, DL-dithiothreitol (DTT), fluorescein diacetate (FDA) and propidium iodide (PI ≥ 94%) were purchased from Sigma-Aldrich (Steinheim, Germany). RPMI 1640, fetal bovine serum (FBS), 1% penicillin/streptomycin, Trypsin-EDTA and Dulbecco's phosphate buffer saline (PBS) were purchased from PAA Laboratories GmbH (Pasching, Austria). 

2-hydroxy-4′-(2-hydroxyethoxy)-2-methylpropiophenone, photoinitiator (PI) Irgacure 2959, PEG-*b*-PPG-*b*-PEG diacrylate and PEG575 diacrylate were purchased from Sigma-Aldrich; 8arm PEG acrylate was purchased from Jenkem technology (Plano, TX, USA). Physicochemical properties of the corresponding block copolymer and PEG derivatives can be found in Table 1. Silicon wafers (polished on one side) were obtained from Microchemicals (Ulm, Germany), and silicon masters were purchased from Amo GmbH (Aachen, Germany). Ultrapure deionized water was used for all solution preparation. All glassware was cleaned with Aqua Regia (*V*_HNO3_:*V*_HCl_ = 1:3).

All chemicals used as received unless stated otherwise. Solvents were of at least analytical grade quality. Ultrapure deionized water was used for all solution preparation.

### 2.2. Instrumental

Silicon masters were made by Amo GmbH in special sizes. The UV lamp (λ = 366 nm, Vilber Lourmat GmbH, (Eberhardzell, Germany) was used for UV curing. SEM measurements were performed on DSM 982 offered by ZEISS Company (Oberkochen, Germany), the optical parts of the microscope from Gemini Optics (Rochester, NY, USA). The hydrogels were carbon coated prior to measurements, which were performed using an Inlens detector operated at 20 KV. Optical images were obtained using a Carl Zeiss fluorescent microscope (Göttingen, Germany). Fluorescence microscopy Axio Observer Z1 (Carl Zeiss, Göttingen, Germany) was used to achieve optical sectioning through the fluorescent sample. Images were taken using an AxioCam MRm digital camera and analyzed using the Axio Vision V4.8.1 software package (Carl Zeiss, Göttingen, Germany).

### 2.3. Preparation of Hydrogels

#### 2.3.1. 3BC-UV Hydrogel

The liquid precursor of block copolymer (3BC with molecular weight 4400) containing 1% of PI (1 wt % with respect to the amount of the precursor) were firstly mixed in a vial. Then the vial was put into oven at 60 °C for about 5 min until the mixture became clear. Subsequently, 80 µL precursor mixtures were deposited on a clean glass slide, capped with a cover glass (18 mm × 18 mm Carl Roth GmbH & Co KG) and exposed to UV light (λ = 366 nm Vilber Lourmat GmbH) for 15 min using a working distance of 10 cm in a nitrogen-filled glovebox. The cured transparent hydrogels were peeled off with tweezers, and then the samples were kept in water in a petri dish.

#### 2.3.2. PEG575 Hydrogel

PEG (with molecular weight 575) liquid precursors containing 1% of PI (1 wt % with respect to the amount of the precursor) were mixed in a vial. Then the vial was put into oven at 60 °C about 5 min until the mixture became clear. Subsequently, 80 µL of the as-prepared mixtures were deposited on a clean glass slide, capped with a cover glass (18 mm × 18 mm Carl Roth GmbH & Co KG) and exposed to UV light (λ = 366 nm Vilber Lourmat GmbH) for 30 min using a working distance of 10 cm in a nitrogen-filled glovebox. The cured transparent hydrogels were peeled off with tweezers, and then the samples were kept in water in a petri dish.

#### 2.3.3. 8PEG-UV Hydrogel

8PEG-UV hydrogel was synthesized by our previously published procedure [31]. Aqueous solutions of 8PEG, (50 wt %) containing 1% of PI (1 wt % with respect to the amount of the precursor) were mixed in a vial. Then the vial was put into oven at 60 °C about 5 min until the mixture became clear. Subsequently, 80 µL of the as-prepared mixtures were deposited on a clean glass slide, capped with a cover glass (18 mm × 18 mm Carl Roth GmbH & Co KG) and exposed to UV light (λ = 366 nm Vilber Lourmat GmbH) for 30 min using a working distance of 10 cm in a nitrogen-filled glovebox. The cured transparent hydrogels were peeled off with tweezers. And then the samples were kept in water in a petri dish. 

#### 2.3.4. Synthesis of 8PEG-VS-SH Hydrogel

Different amounts of ammonium solution (30% NH_3_ in H_2_O) were added to the precursor solution of 8-arm poly(ethylene glycol) vinyl sulfone (8PEG-VS) with 50% water content at room-temperature under vigorous magnetic stirring until the solution turned to a viscous liquid. Compositions were set in order to receive 20%, 10%, 5% and 2.5 wt % NH_3_-8PEG by weight. The resulting liquids were deposited on a glass slide and covered with a glass cover slip. After 30 min, the 8PEG-VS hydrogel were formed. After gel formation, the colorless polymeric films formed with 5% NH_3_ were peeled off mechanically. The stand-alone films (250–300 µm in thickness) were handled with tweezers. These hydrogels were immersed in DTT solution (5 mg/mL) for 60 min. Afterwards, these hydrogels were washed thoroughly with water for several times and stored in water before use.

### 2.4. Deposition of Au NPs on Silicon Wafers

After ultrasonication in a mixture of acetone and water (*v/v* = 1:1) for 20 min, the silicon wafers were immersed in Piranha solution (mixture of H_2_SO_4_ and H_2_O_2_ with *v/v* = 7:3) for 30 min. They were washed thoroughly with Milli-Q water and isopropanol, and then dried under a stream of pure nitrogen gas. Afterwards, the as-prepared silicon wafers were placed inside a small Teflon chamber filled with a solution of APTES (100 µL). APTES was then introduced into the sealed chamber with raising the pressure of the deposition chamber [32]. After 2 h of reaction, the silicon wafers were washed with anhydrous toluene (×3) and isopropanol (×1), and immediately dried with nitrogen followed by evacuation. Then, deposition of Au NPs onto silicon wafers was carried out; a drop of 100 µL homogeneously dispersed Au NPs with diameter 42 nm was placed on APTES modified silicon wafer. After incubation for 60 min, the silicon wafers were washed thoroughly with deionized water for 8 times and then dried with nitrogen gas. They were kept in a glove box to avoid oxidization before use.

### 2.5. Fabrication of Micro-Patterned Block Polymer Hydrogel Replicas Transfer of the Patterned Au NPs from Silicon Wafer to 8PEG-VS-SH Hydrogels

**Figure 1:**
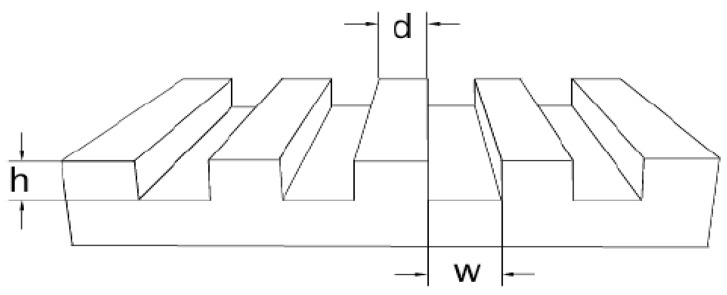
Schematic view of a patterned silicon master.

PEG-PPG-PEG (block polymer, 4400Da, Sigma-Aldrich) replica with micropatterns of lines were prepared by replication from silicon masters (width × distance × height = 20 × 10 × 5, 50 × 10 × 5 µm) as shown in Figure 1, which comprise patterned stripes constructed into microscale lines. Silicon wafers were rinsed with acetone, water, and isopropanol and dried under a mild stream of nitrogen before use. Prior to the replication the cleaned silicon masters were fluorinated with trichloro (1*H*, 1*H*, 2*H*, 2*H*-perfluorooctyl) silane 97% (Sigma-Aldrich, Steinheim, Germany). The viscous liquid of block polymer was dispensed on the silicon master (Figure 1), covered with a thin glass coverslip and exposed to UV light (λ = 366 nm Vilber Lourmat GmbH) for 15 min using a working distance of 10 cm, in a nitrogen-filled glovebox. After crosslinking, the polymeric film was mechanically peeled off from the silicon master by using tweezers. 

### 2.6. Transfer of the Patterned Au NPs from Silicon Wafer to 8PEG-VS-SH Hydrogels

Subsequently, a stand-alone film of the 8PEG-VS-SH hydrogel was placed into conformal contact with the silicon wafers with patterned Au NPs for 30 s. Then, it was peeled off carefully to convey all Au NPs from silicon to the gel surface. The hydrogel was washed for 3 times with deionized water in order to remove any non-adsorbent Au NPs. The final samples were kept in water in swollen state for cell culture, and other samples were kept at room temperature for 12 h in dried state for SEM measurements. 

### 2.7. Cell Culture

Murine fibroblasts L-929 were kindly provided by Dr. J. Lehmann (Fraunhofer Institute for Cell Therapy and Immunology IZI, Leipzig, Geramy). L-929 cells were cultured in 75 cm^2^ cell culture flasks containing RPMI 1640 supplemented with 10% fetal bovine serum (FBS) and 1% penicillin/streptomycin (PS, 100×, all PAA Laboratories GmbH, Pasching, Austria) at 37 °C and 5% CO_2_ in a humidified incubator. The cells were grown until confluence, washed with Dulbecco’s phosphate buffered saline solution and treated with Trypsin-EDTA (PAA Laboratories GmbH). After incubation for 2–5 min at 37 °C, the detached cells were suspended in cell culture medium. The cell suspension was transferred into a falcon tube (VWR International GmbH, Darmstadt, Germany) and centrifuged for 3 min at 1300 rpm, 4 °C. Finally, the cell pellet was resuspended in fresh medium and cells were counted using a hemocytometer (Paul Marienfeld GmbH & Co. KG, Lauda Königshofen, Germany). Cell culture medium was refreshed every second day. The cells were taken out from incubator at 3, 24 or 96 h for taking images in microscopy.

#### Incubation with L-929 Cells

After spraying ethanol (70% *v/v*) on both sides of hydrogels, they were washed carefully by deionized water and waited for drying in the sterile bench. Afterwards, they were put into each 8-well plates with 300 µL of a cell suspension containing 20,000 cells/mL L-929 cells, and incubated at 37 °C, 5% CO_2_ atmosphere and 100% humidity. The adhered cells were detected by optical microscopy after incubation for 3, 24 and 96 h, respectively. 

## 3. Results and Discussion

### 3.1. Characterization of Au NPs Immobilized on the Surface of Hydrogels by UV-Vis Spectroscopy

**Figure 2:**
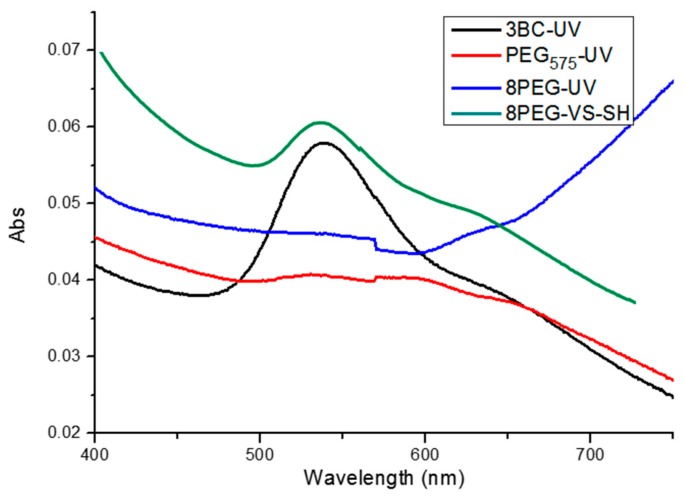
UV-vis spectra of the as-synthesized gold nanocomposite hydrogels.

After the Au NPs had been transferred to the four different hydrogels, the as-obtained nanocomposite hydrogels were characterized by UV-vis spectroscopy as shown in Figure 2. Absorption peaks at 530 nm can be observed for the 3BC-UV and 8PEG-VS-SH hydrogels immobilized Au NPs, which is the characteristic peak of Au NPs [30]. This indicates that the Au NPs have been successfully immobilized on the hydrogel surface, corresponding to the color change of the hydrogels from colorless to red color before and after Au NPs are transferred to the surface of 3BC-UV and 8PEG-VS-SH hydrogels. Correspondingly, the absence of the absorption peak at 530 nm in the spectra of PEG575-UV and 8PEG-UV hydrogels indicates that Au NPs are not successfully transferred to these two hydrogels.

### 3.2. Characterization of Immobilized Au NPs on the Surface of Hydrogels by SEM

In order to investigate the transfer efficiency of Au NPs from silicon wafers to hydrogels via this novel method, SEM was utilized to characterize and count the number of Au NPs on the silicon before and after transferring procedure. It should be noted that during the transfer process the hydrogels were in swollen state, while for the SEM measurements, they were in dried state. Depending on the swelling degree of the different gels, which is the largest for 8PEG gels, the gels as observed by electron microscopy are shrunk down to one fourth of their swollen volume. The particle density therefore seems higher on the (before) more swollen and (afterwards) more shrunk gels. 

**Figure 3:**
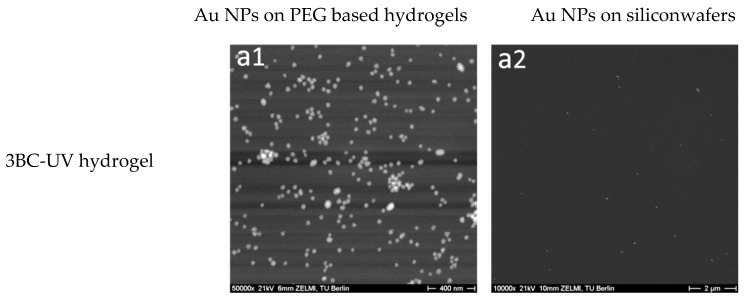

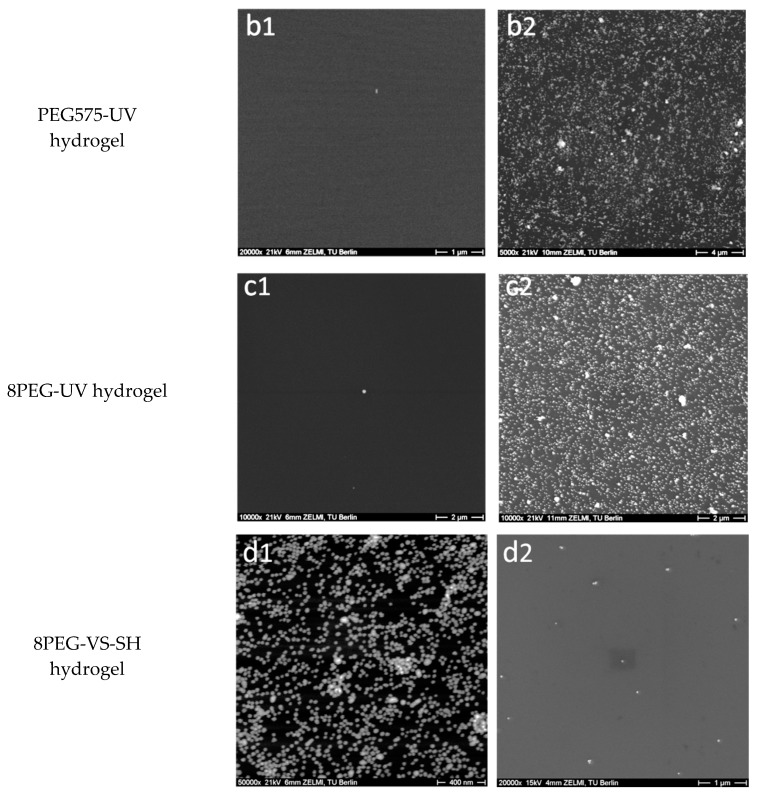
SEM images of Au NPs on the surface of (**a1**) 3BC-UV hydrogels; (**b1**) PEG575-UV hydrogels; (**c1**) 8PEG-UV hydrogels; (**d1**) 8PEG-VS-SH hydrogels and silicon wafers, after the transfer procedure (**a2**–**d2**).

SEM images shown in Figure 3 provide a direct observation for the distribution of Au NPs on the hydrogels. As can be observed from Figure 3a1,d1, a monolayer of Au NPs is present on the surface of 3BC-UV and 8PEG-VS-SH hydrogels after the hydrogels were peeled off from silicon wafers. Only few Au NPs are left on the silicon wafers shown in Figure 3a2,d2. The transfer efficiency of Au NPs from silicon wafers to these hydrogels is 99.8% and 98% (Table 2), respectively, indicating that virtually all Au NPs are transferred from silicon wafers onto the surface of 3BC-UV and 8PEG-VS-SH hydrogels. In contrast, hardly any Au NPs are detected on the PEG575-UV and 8PEG-UV hydrogels shown in Figure 3b1,c1. The transfer efficiency of Au NPs from silicon wafers to these hydrogels is less than 1% (Table 2).

The manifest discrepancy in the transfer efficiency of the different gels is intriguing. In order to understand this, we have to consider several physicochemical properties that differ between the 4 different hydrogels.

We hypothesize that the difference in the transfer efficiency could—at least partly—be due to the different hydrophobicity of these hydrogels. While 3BC-UV, PEG575-UV and 8PEG-UV hydrogels are formed through the same crosslinking method, the chemical structure of 3BC is different from that of the pure PEG-hydrogels PEG575 and 8PEG. The PPG-block in 3BC is much more hydrophobic than PEG-segments, which makes this gel more hydrophobic in comparison to PEG575-UV and 8PEG-UV hydrogels [33].

Besides, the flexibility of the different gels is also expected to play a great role in determining the transfer efficiency. For instance, in comparison with PEG575-UV, 3BC-UV hydrogels are softer, hence more flexible and compliable. Thus, the contact area between 3BC-UV hydrogels and Au NPs modified silicon is therefore larger due to the ease of deformation of 3BC-UV hydrogels. This in turn leads to a higher adhesive force between Au NPs and hydrogels, thereby guaranteeing the Au NPs transfer to the hydrogels. 

Finally, for 8PEG-VS-SH hydrogels, both physical interaction and covalent bonding are responsible for the effective immobilization of Au NPs. The -SH moieties, which covalently attach to the Au NPs, together with the (van der Waals) interaction between Au NPs and flexible PEG polymer chains, both aid the coupling of the Au NPs to the PEG-VS-SH hydrogels. 

**Figure 4:**
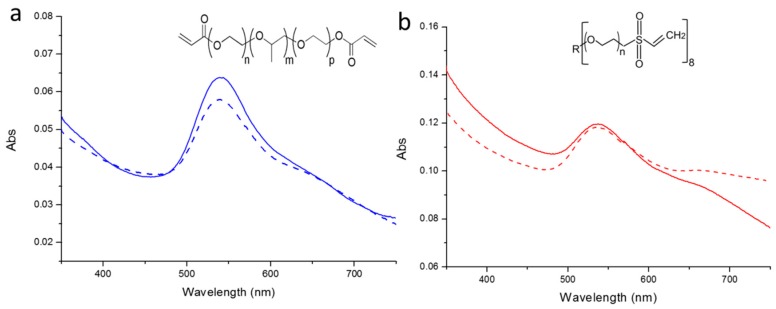
UV-vis spectra of Au NPs on (**a**) 3BC-UV hydrogels and (**b**) 8PEG-VS-SH hydrogels. (solid lines represent Au NPs on the hydrogels before ultrasonication treatment; dashed lines represent Au NPs on the hydrogels after 15 min of ultrasonication treatment).

UV-Vis spectroscopy has been used to characterize the stability of Au NPs bound on the 3BC-UV and 8PEG-VS-SH hydrogels. As shown in Figure 4a,b, an obvious absorption peak at ~530 nm can be observed before and after ultrasonication treatment of Au NPs immobilized on the 3BC-UV and 8PEG-VS-SH hydrogels, which is the characteristic peak of Au NPs. This indicates that Au NPs are firmly immobilized on the hydrogels, and are not easily washed away.

As a result, it can be stated that 3BC-UV and 8PEG-VS-SH-hydrogels can efficiently transfer Au NPs from amino-silanized silicon wafers on their surfaces after contacting of both surfaces. In the further step of the present work, based on these achievements, micro stamps of 3BC are created for deprinting of Au NPs from silicon wafers in order to achieve micro-patterns of Au NPs on the silicon wafers. These patterns of Au NPs are then transferred on 8PEG-VS-SH-hydrogels for studying cellular adhesions, which will be explained in further detail in the following section. 

### 3.3. Synthesis of 8PEG-VS-SH Hydrogel with Patterned Au NPs

**Figure 5:**
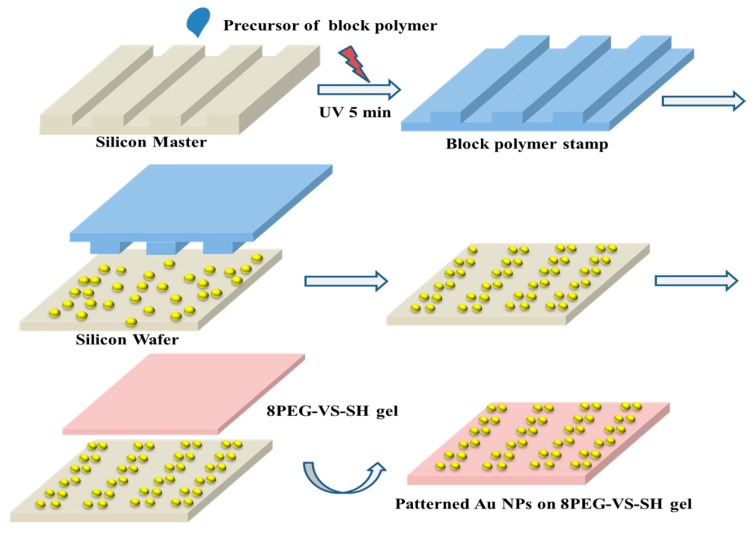
Schematic diagram of patterning Au NPs on the surface of 8PEG-VS-SH hydrogels in the form of micrometer stripes.

The strategy to prepare micropatterns of Au NP arrays on the surface of PEG-VS-SH hydrogels is shown in Figure 5. Firstly, APTES that is rich in amino groups, is chemically bound to the silicon wafer by the Chemical Vapor Deposition (CVD) method. Au NPs are then immobilized on the surface of silicon wafers due to the electrostatic interactions between amino groups and negatively charged Au NPs. As could be observed Figure 3 block polymer hydrogel (3BC) can efficiently transfer Au NPs from silicon wafers. Therefore, a block polymer stamp has been subsequently fabricated and used to create patterned Au NPs stripes on the surface of silicon wafer. Because of the geometry of the block polymer stamp, only from the regions where the stamp directly contacts with the substrate, the Au NPs can be taken off. After peeling off the stamp, the remaining Au NPs thus represent a patterned array on the surface of the silicon wafer. 

At last, the patterned Au NPs are transferred from silica wafers onto the surface of the 8PEG-VS-SH hydrogels. The success of this strategy relies on the fact that the Au-S bond between Au NPs and 8PEG-VS-SH hydrogels is stronger than the electrostatic interactions between positively charged amino groups on silicon wafer and negatively charged citrate-stabilized Au NPs.

Two types of patterned Au NPs on the hydrogels have been obtained by using this approach. Au NPs with different sizes (20 and 42 nm) and silicon masters with different line widths (20 and 50 µm) have been applied in this work. SEM has been used to characterize the as-obtained patterned structure on hydrogels. 

**Figure 6:**
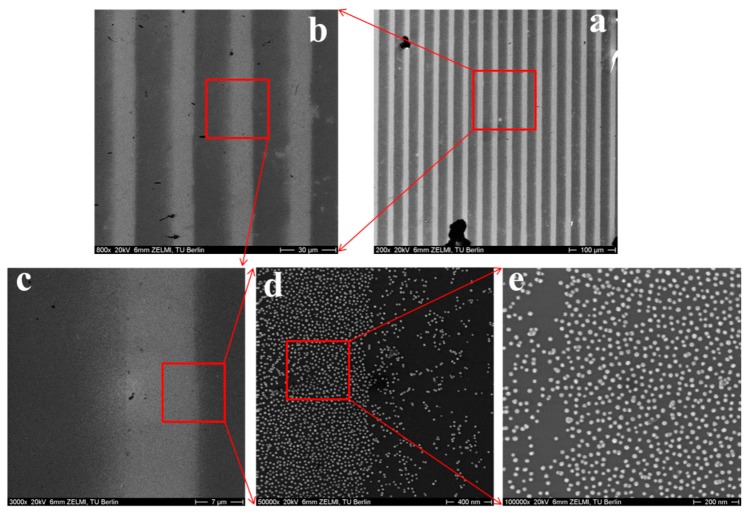
SEM images of patterned Au NPs on the surface 8PEG-VS-SH hydrogels. Scale bar (**a**) 100 µm; (**b**) 30 µm; (**c**) 7 µm; (**d**) 400 nm; (**e**) 200 nm. The size of Au NPs is 20 nm, and the distance between patterned stripes is 20 µm in the swollen state.

When Au NPs with 20 nm in diameter and silicon wafers with 20 µm in width are used, patterned Au NPs stripes are formed on the hydrogels, which can be recognized as straight grey lines from Figure 6c. These patterned Au NPs stripes consist of a densely packed Au NPs without formation of multilayers (Figure 6d,e).

**Figure 7:**
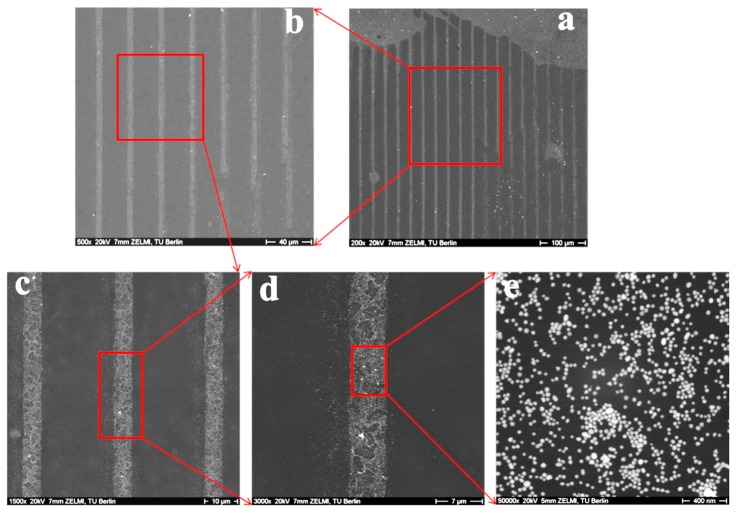
SEM images of patterned Au NPs on the surface of 8PEG-VS-SH hydrogels. Scale bar (**a**) 100 µm; (**b**) 40 µm; (**c**) 10 µm; (**d**) 7 µm; (**e**) 400 nm. The size of Au NPs is 42 nm, the width of stripe is 10 µm and the distance between patterned stripes is 50 µm in the swollen state.

When Au NPs with 42 nm in diameter and silicon wafers with 50 µm lines are used, similar results are obtained. Straight grey lines with lines of 10 µm in width (distance between stripes is 50 µm) can be recognized from Figure 7a–c, indicating that patterned Au NPs stripes are formed on the hydrogels. At higher magnification, it can be seen how the Au NPs are distributed on the hydrogels; not perfectly homogeneously, but large agglomerations are not present either.

### 3.4. Cell Adhesion and Cell Spreading

**Figure 8:**
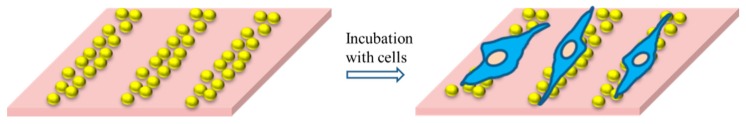
General scheme of the patterned Au NPs stripes on the surface of 8PEG-VS-SH hydrogels to control cell adhesion and spreading.

Cell adhesion tests were carried out on these patterned Au NPs-hydrogel-nanocomposites. Although the PEG-based hydrogel background is supposed to be anti-adhesive, and the Au NPs are not (bio)functionalized to assist cell adhesion, we know from our very recent research that cells do adhere to these nanocomposite surfaces; even more than on tissue culture polystyrene (TCPS) controls [14]. Consequently, in this work, controlled cell adhesion on Au NPs micro-stripes was expected (see Figure 8).

We have considered the effects of (surface) chemistry, topography and elasticity to explain the cell adhesion to the nanocomposites. First of all, it is obvious that the gold surface of the nanoparticles is not as anti-adhesive as the PEG-gel background. This could be due to serum proteins adsorbing to the gold surface and providing anchoring points for cells to adhere. Second, the presence of nanostructures might give the cells some handles to adhere to. Third, the soft hydrogel allows the diffusion of proteins into the substrate, by which the interface may become enriched with proteins, some of which may assist cell adhesion.

Cell adhesion process comprises three stages: attachment, spreading, and formation of focal adhesions and stress fibers [34,35]. At the first stage, cells are very sensitive to the environmental conditions. Any variation in the environment will result in their attachment or detachment from the substrate [36,37]. For instance, the variation of the rigidity properties and surface roughness correlates with the changes of cell adhesion [38,39]. 

**Figure 9:**
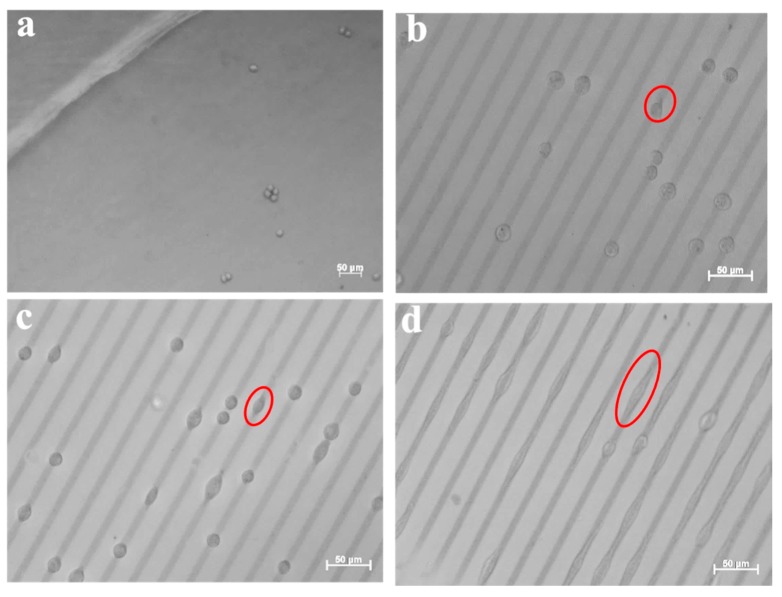
Optical images of L-929 cells after incubation with patterned Au NPs (d = 20 nm) on the surface of hydrogels (20 µm inter-stripe distances) at different incubation times: (**a**) pure PEG gel (8PEG-VS-SH); (**b**) 3 h; (**c**) 24 h and (**d**) 48 h, respectively. Red ellipses refer to L-929 cell growing on the patterned Au NPs stripes.

In order to investigate the influence of the incubation time on the cell adhesion and spreading, different incubation times have been applied. The cellular behavior of L-929 cells on the surface of 8PEG-VS-SH hydrogels with patterned 20 nm Au NPs stripes (10 µm in width and 20 µm in distance) had been firstly investigated. In the optical image in Figure 9, the L-929 cells tend to adhere to the patterned Au NPs stripes already after incubation for 3 h (Figure 9b), whereas hardly any cells on the surface of the pure hydrogel without Au NPs are seen (Figure 9a). With increasing the incubation time to 24 h, the cells start to spread along the patterned Au NPs stripes (Figure 9c). After incubation for 48 h, cells cover the whole pattern lines (Figure 9d), while the morphology of cells changes from round to spindle-like, indicating that the patterned Au NPs on 8PEG-VS-SH hydrogels have a great impact on the murine fibroblasts L-929 cell adhesion and spreading.

**Figure 10:**
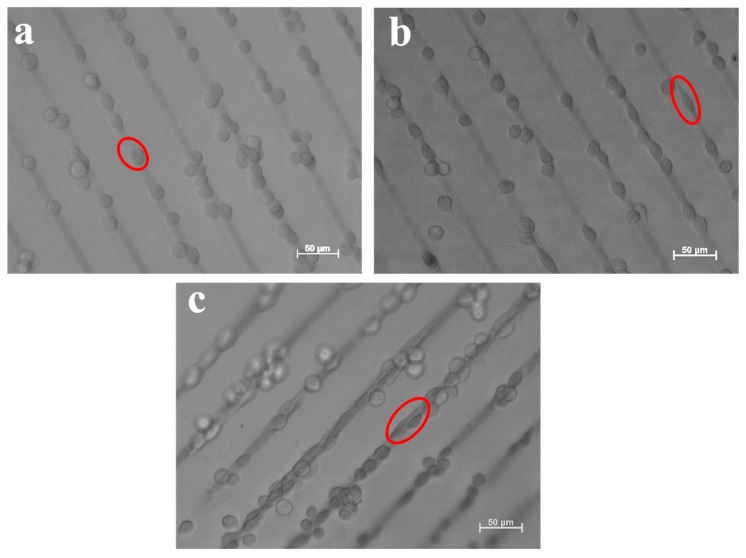
Optical images of L-929 cells incubated with patterned Au NPs (d = 42 nm) on the surface of hydrogels (50 µm in distance of the inter-stripes) for different incubation time: (**a**) 3 h; (**b**) 24 h and (**c**) 48 h, respectively. Red ellipses refer to L-929 cell growing on the patterned Au NPs stripes.

The cellular behavior of L-929 cells on the surface of 8PEG-VS-SH hydrogels with patterned 42 nm Au NPs stripes (10 µm in width and 50 µm in distance) had been further investigated. Figure 10a shows that the cells adhere to the patterned Au NPs lines after 3 h of incubation. This phenomenon is distinctly different from L-929 cells cultured on TCPS, where the cells grow with random distribution. Similarly, the cells tend to grow along the direction of the patterned Au NPs stripes after incubation for 24 h (Figure 10b). With increasing the incubation time to 48 h, cell proliferation leads to a slight increase in cell number. Cells agglomerate and form clusters consisting of several individual cells along the direction of the patterned Au NPs stripes (Figure 10c). The patterned Au NPs stripes, indeed, induce cell alignment and promotes cell adhesion, spreading and proliferation along the patterned direction. 

The result demonstrates that the patterned Au NPs stripes can be transferred to the 8PEG-VS-SH hydrogel to promote cell adhesion and spreading in an ordered way. After incorporation of Au NPs onto the surface of hydrogels, an increase in the stiffness and roughness of composite hydrogels may induce cell adhesion. In addition, surface topographies in the micrometer and nanometer range may also induce cell adhesion. The present study shows that the patterned Au NPs stripes can be used for controlling a cellular response, and affect the morphology, adhesion of the fibroblasts cells. More importantly, patterning cells on the substrate is useful for the development of tissue engineering and fundamental studies in cell biology.

### 3.5. Cell Viability

**Figure 11:**
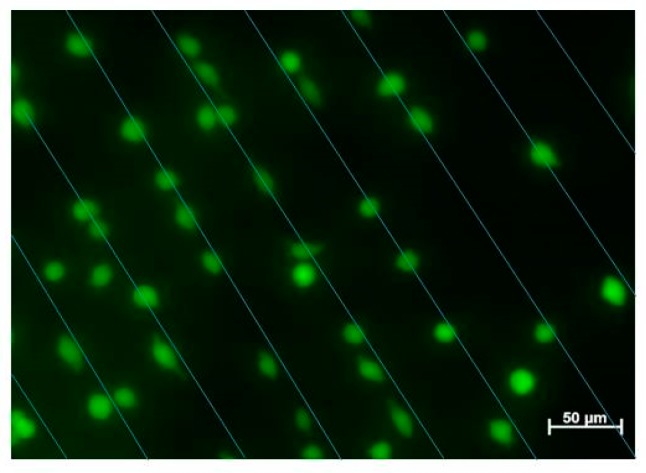
Fluorescent images of L-929 cells after being cultured on the surface of 8PEG-VS-SH hydrogel with patterned Au NPs (d = 42 nm) stripes (50 µm in distance of the inter-stripes) for 24 h. Straight lines were drawn to represent the patterned Au NPs below the cells.

Live/dead staining assay has been used to study the cell viability after incubation with patterned Au NPs stripes for a certain time. In Live/dead staining assay, dead cells appear red, whereas living cells appear green when observed with a fluorescence microscope. As shown in Figure 11, all the cells appear to be green, indicating that the patterned Au NPs stripes are non-cytotoxic to L-929 cells. This is due to the fact that all the Au NPs are firmly immobilized on the surface of hydrogels without entering the cells.

## 4. Conclusions

In this study, after uniform deposition of Au NPs on the surface of silicon wafers 3BC-UV, PEG575-UV, 8PEG-UV and 8PEG-VS-SH hydrogels have been employed to immobilize Au NPs by transferring them from silicon wafers to the surface of the hydrogels. SEM analysis have revealed that that 3BC-UV and 8PEG-VS-SH hydrogels can efficiently immobilize Au NPs, and the transfer efficiency is as high as 98%. In contrary, PEG575-UV and 8PEG-UV hydrogels cannot immobilize any Au NPs.

Considering that the 8PEG-VS-SH hydrogel is cell-repellent, and the Au NPs can be efficiently immobilized on the hydrogel surface, these composite hydrogels are applied as substrates for cell culture, and for the investigation of the interactions between Au NPs and cells. Therefore, we develop a soft and stable substrate for efficient immobilization of Au NPs.

Furthermore a simple, time-saving and cost-effective protocol has been developed to create micropatterned Au NPs on the surface of functional PEG hydrogel. This “micro-contact deprinting method” provides a new way to pattern Au NPs onto hydrogel. On this hydrogel with patterned Au NPs, L-929 cells can adhere to and spread on the patterned Au NPs stripes in an ordered way. Therefore, these cyto-compatible hydrogels with patterned Au NPs stripes can be applied for controlling cell adhesion, and they are a very promising candidate for applications in tissue engineering.

## Figures and Tables

**Table 1:** Physicochemical properties of PEG-based precursors: PEG-PPG-PEG (3BC), PEG Diacrylate (PEG), 8arm PEG acrylate (8PEG) and 8arm PEG Vinyl Sulfone (8PEG-VS). Values are obtained from the manufacturer. R: hexaglycerin core structure, r.t: room temperature.

Material	PEG-*b*-PPG-*b*-PEG Diacrylate (3BC)	PEG Diacrylate (PEG)	8-arm PEG Acrylate (8PEG)	8-arm PEG Vinyl Sulfone Acrylate (8PEG-VS)
Structure	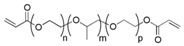	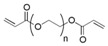	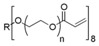	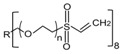
M_w_ [Da]	4400	575	15000	15000
Chain Length	n + p ~12; m ~57	n ~13	n ~40	n ~40
PEG [%]	30 (70 % PPG)	100	100	100
State at r.t.	Liquid	Liquid	Solid	Liquid
Gel formation	UV	UV	UV	Michael Addition Reaction

**Table 2:** Transfer efficiency of Au NPs from silicon wafers to 4 different hydrogels.

Hydrogels	Transfer efficiency (%)
3BC-UV	99.8 ± 0.1
PEG_575_-UV	0
8PEG-UV	0
8PEG-VS-SH	98 ± 1.8
